# Analysis of the influence of memory content of auditory stimuli on the memory content of EEG signal

**DOI:** 10.18632/oncotarget.11234

**Published:** 2016-08-11

**Authors:** Hamidreza Namazi, Reza Khosrowabadi, Jamal Hussaini, Shaghayegh Habibi, Ali Akhavan Farid, Vladimir V. Kulish

**Affiliations:** ^1^ School of Mechanical and Aerospace Engineering, Nanyang Technological University, Singapore; ^2^ Institute for Cognitive and Brain Science, Shahid Beheshti University, Tehran, Iran; ^3^ Faculty of Medicine, Universiti Teknologi MARA, Sungai Buloh, Selangor, Malaysia; ^4^ School of Medicine, Fasa University of Medical Sciences, Fasa, Iran; ^5^ Faculty of Engineering and Technology, Multimedia University, Melaka, Malaysia

**Keywords:** auditory stimulus, electroencephalogram (EEG) signal, memory, Hurst exponent, approximate entropy

## Abstract

One of the major challenges in brain research is to relate the structural features of the auditory stimulus to structural features of Electroencephalogram (EEG) signal. Memory content is an important feature of EEG signal and accordingly the brain. On the other hand, the memory content can also be considered in case of stimulus. Beside all works done on analysis of the effect of stimuli on human EEG and brain memory, no work discussed about the stimulus memory and also the relationship that may exist between the memory content of stimulus and the memory content of EEG signal. For this purpose we consider the Hurst exponent as the measure of memory. This study reveals the plasticity of human EEG signals in relation to the auditory stimuli. For the first time we demonstrated that the memory content of an EEG signal shifts towards the memory content of the auditory stimulus used. The results of this analysis showed that an auditory stimulus with higher memory content causes a larger increment in the memory content of an EEG signal. For the verification of this result, we benefit from approximate entropy as indicator of time series randomness. The capability, observed in this research, can be further investigated in relation to human memory.

## INTRODUCTION

During years analysis of the influence of different types of external stimuli on human brain has been one of the main topics in brain research. For this purpose, scientists mapped the brain reaction using different scanning methods [1] and then analyzed this reaction. Electroencephalogram (EEG) is one of the famous methods which maps the brain activity versus time. A lot of research have been reported which employed different mathematical and computational methods for analysis of EEG signal due to external stimuli [2-6].

The concept of fractal processes has been considered as a useful approach for studying the scaling properties of different time series. The long range correlation is a characteristic of fractal time series, which means the fluctuations, are related to earlier fluctuations. This correlation defines the presence of memory.

One of the exponents that is widely used in fractal theory is the Hurst exponent. The Hurst exponent that is widely used for analysis of fractal time series indicates the memory of the process. The value of the Hurst exponent can be between 0 and 1, where H = 0.5 stands for a truly random process (e.g., Brownian motion).

There has been variety of works in biology and medicine which employed the Hurst exponent for their investigation. Using Hurst exponent in investigation about DNA [7], human gait [8], heart rate [9], heart sound [10] and eye movement [11] are noteworthy to mention. In case of fractal EEG signals, beside some works that analyzed the Hurst exponent for EEG signals without any external stimulation [12-14], very limited works investigated the variation of the Hurst exponent for EEG signal due to external stimulation. For instance, in [15] we showed that the value of EEG signal's Hurst exponent increases due to visual stimulation. In that paper we also developed a mathematical model of an EEG signal in the form of power law. Focusing on employing auditory stimulus, we can report the work done by Dey et al [16]. They employed Detrended Fluctuation Analysis (DFA) technique to analyze variations of the Hurst exponent for subjects who listen to music. In another work, Natarajan et al. [17] analyzed the EEG signal's Hurst exponent due to music and reflexological stimulation. Their results showed that the value of the Hurst exponent increases from H = 0.5 due to stimulation, which means that the randomness decreases due to music/reflexology. In fact, in all these works it has been stated that the value of the Hurst exponent for the EEG signal increases due to stimulation.

Beside all works done on analysis of the effect of external stimuli on human EEG and memory, no work discussed about the stimulus memory and also relationships that may exist between the memory content of a stimulus and the memory content of an EEG signal. In this research, we hypothesize that the memory content of auditory stimuli should affect the memory content of the corresponding EEG signal. For this purpose, we analyze the relationship between the variation of the Hurst exponent of auditory signal (stimulus) and the variation of the Hurst exponent of EEG signal. For the verification purpose, we employ approximate entropy in order to analyze the randomness of both auditory stimulus and EEG signal.

## RESULTS

We checked the governed data from the subjects. It is noteworthy to mention here that we considered all the collected data for analysis.

**Figure 1:**
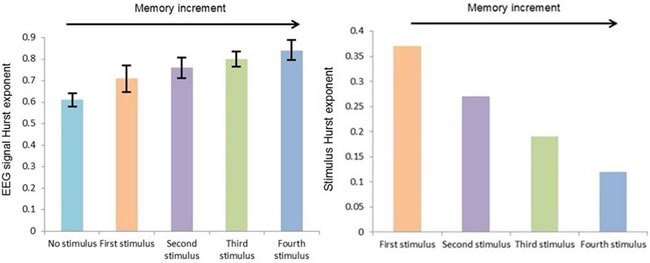
EEG signal's Hurst exponent in case of different auditory stimuli in the range of H < 0.5 Error bars are standard deviations.

Mauchly's test indicated that the assumption of sphericity had not been violated in case of the outcomes (the Hurst exponent and approximate entropy of EEG signals). Figure 1 shows the variations of mean of EEG signal's Hurst exponent in case of different auditory stimuli in the range of H < 0.5.

Considering *F_crit_* (4,195) = 2.41 at α = 0.05, based on Table 1 the result of statistical analysis [*F*(4,195) = 172.5, *p* = 0.001] indicates that there was a significant effect of auditory stimuli on the Hurst exponent of EEG signals, with an effect size ω^2^ = 0.74. In general, the application of the auditory stimulus increased the Hurst exponent of the corresponding EEG signal.

**Table 1:** The result of ANOVA test in case of EEG signal’s Hurst exponent (95% confidence interval) for the first set of stimuli (H < 0.5)

	SS	df	MS	F	p
**Between**	1.379	4	0.345	172.5	0.001
**Within**	0.460	195	0.002		
**Total**	1.839	199			

A significant linear trend between auditory stimulus conditions was observed (*p* = 0.001), indicating that the fourth stimulus caused a larger increment in the Hurst exponent of an EEG signal than the third stimulus, followed by the second stimulus and the first stimulus. As in all cases, the value of the Hurst exponent is larger than 0.5, it can be said that the fourth stimulus caused a larger memory increment in the EEG signal than the third stimulus, followed by the second stimulus and the first stimulus, reflecting the trend in the memory content of the auditory stimuli i.e. the fourth stimulus (H = 0.12) has a larger memory content than the third stimulus (H = 0.19), which itself has a larger memory content than the second stimulus (H = 0.27), which itself has a larger memory content than the first stimulus (H = 0.37). The effect size calculations between different conditions show that the fourth stimulus led to the greatest change in the Hurst exponent of an EEG signal observed across all fourth stimuli comparisons (Table 2).

**Table 2:** Effect sizes in analysis of EEG signal’s Hurst exponent and approximate entropy for the first set of stimuli (H < 0.5)

Condition	Hurst exponent Effect size (*r*)	Approximate entropy Effect size (*r*)
No stimulus *vs*. First stimulus	0.72	0.55
No stimulus *vs*. Second stimulus	0.87	0.81
No stimulus *vs*. Third stimulus	0.93	0.85
No stimulus *vs*. Fourth stimulus	0.94	0.89
First stimulus *vs*. Second stimulus	0.41	0.43
First stimulus *vs*. Third stimulus	0.66	0.61
First stimulus *vs*. Fourth stimulus	0.76	0.74
Second stimulus *vs*. Third stimulus	0.40	0.39
Second stimulus *vs*. Fourth stimulus	0.62	0.64
Third stimulus *vs*. Fourth stimulus	0.40	0.35

**Figure 2:**
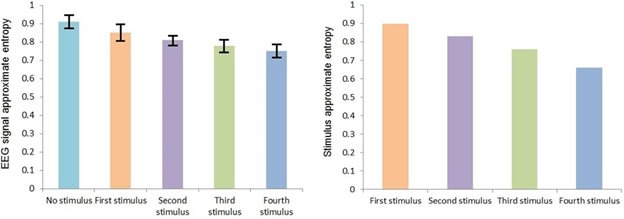
EEG signal's approximate entropy in case of different auditory stimuli in the range of H < 0.5 Error bars are standard deviations.

Figure 2 shows the variations of mean of EEG signal's approximate entropy in case of different auditory stimuli in the range of H < 0.5.

Considering *F_crit_* (4,195) = 2.41 at α = 0.05, based on Table 3 the result of statistical analysis [*F*(4,195) = 78, *p* = 0.001] indicates that there was a significant effect of auditory stimuli on approximate entropy of EEG signals, with an effect size ω^2^ = 0.65. In general, the application of the auditory stimulus decreased the approximate entropy of the corresponding EEG signal.

**Table 3:** The result of ANOVA test in case of EEG signal’s approximate entropy (95% confidence interval) for the first set of stimuli (H < 0.5)

	SS	df	MS	F	*p*
**Between**	0.624	4	0.156	78	0.001
**Within**	0.320	195	0.002		
**Total**	0.944	199			

A significant linear trend between auditory stimulus conditions was observed (*p* = 0.003), indicating that the fourth stimulus caused a larger decrement in the approximate entropy of an EEG signal than the third stimulus, followed by the second stimulus and the first stimulus, reflecting the trend in the approximate entropy of the auditory stimuli i.e. the fourth stimulus has a lower approximate entropy than the third stimulus, which itself has a lower approximate entropy than the second stimulus, which itself has a lower approximate entropy than the first stimulus. The effect size calculations between different conditions show that the fourth stimulus led to the greatest change in the approximate entropy of an EEG signal observed across all fourth stimuli comparisons (Table 2).

In fact, these results agree with the result of analysis of the Hurst exponent because as was mentioned before, approximate entropy is the indicator of randomness of time series, where its smaller value stands for less randomness. So, as we move from the first stimulus to the forth stimulus and the value of the Hurst exponent gets far from H = 0.5, randomness decreases and accordingly approximate entropy will have smaller value. This behavior also can be seen in case of EEG signal randomness and accordingly approximate entropy.

**Figure 3:**
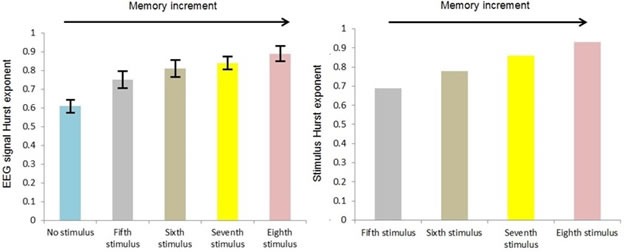
EEG signal's Hurst exponent in case of different auditory stimuli in the range of 0.5 < H Error bars are standard deviations.

Figure 3 shows the variations of mean of EEG signal's Hurst exponent in case of different auditory stimuli in the range of 0.5 < H.

Considering *F_crit_* (4,195) = 2.41 at α = 0.05, based on Table 4 the result of statistical analysis [*F*(4,195) = 249.5, *p* = 0.001] indicates that there was a significant effect of auditory stimuli on the Hurst exponent of EEG signals, with an effect size ω^2^ = 0.83. In general, the application of the auditory stimulus increased the Hurst exponent of the corresponding EEG signal.

**Table 4:** The result of ANOVA test in case of EEG signal’s Hurst exponent (95% confidence interval) for the second set of stimuli (0.5 < H)

	SS	df	MS	F	*p*
**Between**	1.995	4	0.499	249.5	0.001
**Within**	0.382	195	0.002		
**Total**	2.377	199			

A significant linear trend between auditory stimulus conditions was observed (*p* = 0.001), indicating that the eighth stimulus caused a larger increment in the Hurst exponent of an EEG signal than the seventh stimulus, followed by the sixth stimulus and the fifth stimulus. As in all cases, the value of the Hurst exponent is larger than 0.5, it can be said that the eighths stimulus caused a larger memory increment in the EEG signal than the seventh stimulus, followed by the sixth stimulus and the fifth stimulus, reflecting the trend in the memory content of the auditory stimuli i.e. the eighth stimulus (H = 0.93) has a larger memory content than the seventh stimulus (H = 0.86), which itself has a larger memory content than the sixth stimulus (H = 0.78), which itself has a larger memory content than the fifth stimulus (H = 0.69). The effect size calculations between different conditions show that the eighth stimulus led to the greatest change in the Hurst exponent of an EEG signal observed across all fourth stimuli comparisons (Table 5).

**Table 5:** Effect sizes in analysis of EEG signal’s Hurst exponent and approximate entropy for the second set of stimuli (0.5 < H)

Condition	Hurst exponent Effect size (*r*)	Approximate entropy Effect size (*r*)
No stimulus *vs*. Fifth stimulus	0.85	0.74
No stimulus *vs*. Sixth stimulus	0.91	0.88
No stimulus *vs*. Seventh stimulus	0.94	0.94
No stimulus *vs*. Eighth stimulus	0.96	0.95
Fifth stimulus *vs*. Sixth stimulus	0.51	0.48
Fifth stimulus *vs*. Seventh stimulus	0.70	0.77
Fifth stimulus *vs*. Eighth stimulus	0.83	0.83
Sixth stimulus *vs*. Seventh stimulus	0.31	0.57
Sixth stimulus *vs*. Eighth stimulus	0.66	0.74
Seventh stimulus *vs*. Eighth stimulus	0.52	0.49

**Figure 4:**
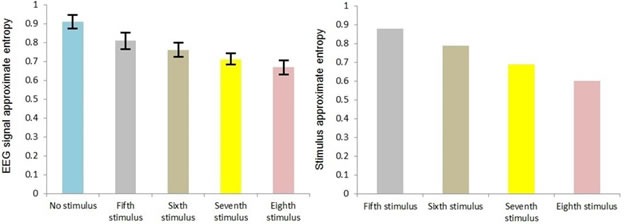
EEG signal's approximate entropy in case of different auditory stimuli in the range of 0.5 < H Error bars are standard deviations.

Figure 4 shows the variations of mean of EEG signal's approximate entropy in case of different auditory stimuli in the range of 0.5 < H.

Considering *F_crit_* (4,195) = 2.41 at α = 0.05, based on Table 6 the result of statistical analysis [*F*(4,195) = 174.5, *p* = 0.001] indicates that there was a significant effect of auditory stimuli on approximate entropy of EEG signals, with an effect size ω^2^ = 0.80. In general, the application of the auditory stimulus decreased the approximate entropy of the corresponding EEG signal.

**Table 6:** The result of ANOVA test in case of EEG signal’s approximate entropy (95% confidence interval) for the second set of stimuli (0.5 < H)

	SS	df	MS	F	*p*
**Between**	1.395	4	0.349	174.5	0.001
**Within**	0.320	195	0.002		
**Total**	1.715	199			

A significant linear trend between auditory stimulus conditions was observed (*p* = 0.001), indicating that the eighth stimulus caused a larger decrement in the approximate entropy of an EEG signal than the seventh stimulus, followed by the sixth stimulus and the fifth stimulus, reflecting the trend in the approximate entropy of the auditory stimuli i.e. the eighth stimulus has a lower approximate entropy than the seventh stimulus, which itself has a lower approximate entropy than the sixth stimulus, which itself has a lower approximate entropy than the fifth stimulus. The effect size calculations between different conditions show that the eighth stimulus led to the greatest change in the approximate entropy of an EEG signal observed across all eighth stimuli comparisons (Table 5).

In fact, these results agree with the result of analysis of the Hurst exponent because as was mentioned before, approximate entropy is the indicator of randomness of time series, where its smaller value stands for less randomness. So, as we move from the fifth stimulus to the eighth stimulus and the value of the Hurst exponent gets far from H = 0.5, randomness decreases and accordingly approximate entropy will have smaller value. This behavior also can be seen in case of EEG signal randomness and accordingly approximate entropy.

In general, it can be concluded that an auditory stimulus with higher memory content (lower approximate entropy) has a stronger effect on the increment of EEG signal's memory. In fact, this investigation, for the first time, shows that there is a coupling between the memory content of an auditory stimulus and the memory content of the corresponding EEG signal.

## DISCUSSION

In this paper, for the first time, we analyzed the influence of the memory content of auditory stimuli on the memory content of an EEG signal. Our results demonstrated plasticity of the EEG signal in relation to the auditory stimuli, as the trend across the memory content of auditory stimuli is reflected in the trend across the memory content of EEG signals. We found out that an auditory stimulus with higher memory content causes a larger increment in the memory content of the EEG signal compared to another auditory stimulus that has less memory content. This result was verified by analyzing the approximate entropy of both auditory stimulus and EEG signal, where the auditory stimulus with lower value of approximate entropy (less randomness) brings the approximate entropy of EEG signal lower, thus making it less random.

Further investigation on the result of this research may explain the observation of scientists about the effect of music on human memory. For instance, Söderlund et al. [18] have demonstrated that adding an auditory white noise (WN) to the environment enhanced the memory performance of children with ADHD-type problems. See also [19]. Please note that here we don’t want to directly link auditory signal's memory and human memory, as this point needs to be discovered more.

There has been a variety of research that investigated the influence of auditory stimuli on the brain of patients with some neurological disorders [20-22]. In this way, our method can be further investigated in case of patients with different brain diseases to improve their brain reaction. For instance, we can use our method in case of patients with Alzheimer in order to investigate how much the auditory stimulus memory content can affect corresponding EEG signals and accordingly the patient's memory.

Also, this investigation can be done in case of other brain status (for instance, during sleep), in order to analyze the effect of auditory stimulation on EEG signal's memory. This analysis may provide the answer to some questions such as “why sound stimulation can improve sleep?” [23].

On the other hand, our analyses would guide on-going efforts to develop realistic models of the brain response to external stimuli. For instance, the result of analysis in this paper can be coupled with our model presented for the brain response to external stimuli in [15] to mathematically write the relations investigated in this research.

In general, increased understanding of the relationship between an auditory stimulus and the brain response shall speed up different research on the analysis of the brain reaction.

## MATERIALS AND METHODS

In this research we aim to study the effect of the memory content of an auditory stimulus on the memory content of an EEG signal. For this purpose we employ the Hurst exponent as the measure of memory.

The Hurst exponent that is discussed in case of analysis of time series can have any value between 0 and 1. H = 0.5 stands for Brownian motion. In this condition the process has no persistence at all, i.e., the probability of the process to continue in the same direction as in the previous step equals the probability of the process changing its direction. Hence, there is no memory of the past at all. For H < 0.5, the probability of the process to continue in the same direction as in the previous step is less than the probability of changing direction. Hence, the process is anti-persistent. On the other hand, for H > 0.5, the process continues in the same direction as in the previous step with the probability larger than the probability of changing direction. Hence, the process is persistent.

Now, considering the concept of memory, H = 0.5 stands for no memory in the process; H = 0 or 1 stands for absolute memory. So, as the value of H is closer to 0.5, it can be said that the process in question has less memory. In other words, the larger the absolute value of (H - 0.5) the stronger the memory of the process is. For instance, H = 0.2 stands for higher memory than H = 0.3. Also, H = 0.8 stands for higher memory than H = 0.6. Thus, in analysis of the Hurst exponent with respect to memory, beside considering the value of the Hurst exponent, we also should pay attention to the range that the Hurst exponent falls within, 0 < H < 0.5 or 0.5 < H < 1.

We also analyze the randomness of both auditory stimulus and EEG signal using approximate entropy. Approximate entropy is the indicator of randomness of time series, where its smaller value stands for less randomness. Thus, as the value of H is closer to H = 0.5, we should have the larger value for approximate entropy.

For our experiments we chose two sets of auditory stimuli with different values of the Hurst exponent (and accordingly approximate entropy) as auditory stimuli. The first set contains four pink noises with the Hurst exponent in the range of H < 0.5. The second set of stimuli contains four music with embedded black noises that have the Hurst exponent in the range of 0.5 < H.

We play each stimulus for subjects and then we analyze the influence of these stimuli on the EEG signal by computing and investigating the Hurst exponent between them. The result of the variation in the Hurst exponent for the EEG signal will be discussed in relation with the variation in the Hurst exponent for the auditory stimuli. The result will also be discussed from the aspect of memory and randomness.

### Data collection

The data collection was done on 40 voluntary healthy subjects (20 M and 20 F) within the age range between 20 and 22 years old. A physician interviewed each subject prior to the experiment, to ensure no hearing problem, neurological deficit, pain condition, or medication affects the EEG data collection. Also, it should be noted that subjects didn’t drink alcohol or other beverages (containing caffeine) that can affect the EEG data for more than 48 hours before data collection. It is noteworthy that all procedures were approved by the Internal Review Board of the university, and the written informed consent was obtained from subjects, after we explained the study to them.

In order to insulate the subjects we have done the experiments in an electrically shielded, acoustically isolated, and dimly illuminated. We instructed the subjects to focus on the auditory stimulus without doing any movement, while they sit comfortably. Also, they were asked to not think to anything.

As was mentioned before, we used two sets of auditory stimuli. The first set contains four pink noises with different values of the Hurst exponent (and accordingly approximate entropy) as auditory stimuli in the range of H < 0.5. In order to generate a pink noise, first, we create a white noise we used rand () function of MATLAB. The pink noise was obtained from the white noise by low-pass filtering. All noises were set below 75 db to avoid un-comfortable hearing condition for the subjects.

As can be seen in Table 7, the Hurst exponent for the first stimulus (H = 0.37) is larger than for the second stimulus (H = 0.27), which itself is larger than for the third stimulus (H = 0.19), which itself is larger than for the fourth stimulus (H = 0.12). As was mentioned before, the value of the Hurst exponent that is closer to 0.5 indicates a lower memory content. Thus, the first stimulus has a lower memory content than the second stimulus, which itself has a lower memory content than the third stimulus, which itself has a lower memory content than the fourth stimulus. In case of approximate entropy, as we move from the first to the forth stimulus, the randomness decreases and accordingly approximate entropy decreases.

**Table 7:** The Hurst exponent and approximate entropy for the first set of stimuli (H < 0.5)

No.	Hurst exponent	Approximate entropy
1	0.37	0.90
2	0.27	0.83
3	0.19	0.76
4	0.12	0.66

In case of the second set of stimuli, we had four music with 0.5 < H. In generation of the music, we embedded four different black noises into a well-known melody by manipulating the inter-beat interval. It is noteworthy to mention that all the music was generated using Musical Instrument Digital Interface files so that the onset of each note can be designed precisely and the inter-beat interval dynamics can be controlled.

As can be seen in Table 8, the Hurst exponent for the eighths stimulus (H = 0.93) is larger than for the seventh stimulus (H = 0.86), which itself is larger than for the sixth stimulus (H = 0.78), which itself is larger than for the fifth stimulus (H = 0.69). As was mentioned before, the value of the Hurst exponent that is closer to 0.5 indicates a lower memory content. Thus, the fifth stimulus has a lower memory content than the sixth stimulus, which itself has a lower memory content than the seventh stimulus, which itself has a lower memory content than the eighth stimulus. In case of approximate entropy, as we move from the fifth to the eighth stimulus, the randomness decreases and accordingly approximate entropy decreases.

**Table 8:** The Hurst exponent and approximate entropy for the second set of stimuli (0.5 < H)

No.	Hurst exponent	Approximate entropy
5	0.69	0.88
6	0.78	0.79
7	0.86	0.69
8	0.93	0.60

The generated noise/music was played using digital voice recorder and music player (Olympus WS-321M) and then transferred to subjects using earphones (Philips SHE1360/97). During data collection, we asked the subjects about their condition, and no one was uncomfortable with the stimuli.

**Figure 5:**
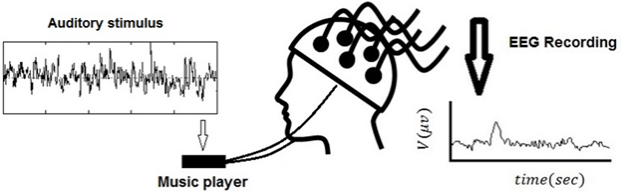
A schematic of the experiment

The EEG data (with sampling frequency of 256 Hz) were collected using Mindset 24 device. The electrode impedance was kept lower than 5KΩ. In the first round, the data collection was done without any stimulus. After that in order to test the effect of an auditory stimulus, we played the first stimulus for the subject for 1 min and collected the EEG signal. After finishing the data collection for the first stimulus we waited for 5 min and then we presented the second stimulus to the subject with the same procedure and continued to test all stimuli. A bipolar electrooculogram (EOG, vertical and horizontal) was recorded to reject off-line artifacts. A schematic of the experiment is shown in Figure 5.

To examine the reproducibility of the results from experiments, the data collections were repeated in the second day for each subject. In total, two trials were collected in case of each stimulus from each subject. A physician monitored all steps of the experiments.

### Data analysis

Since the recorded EEG data were noisy, first these data were filtered using Wavelet toolbox in MATLAB and then were processed for computing of the Hurst exponent and approximate entropy. In this research the analysis was done on the data governed from the left-side temporal (T3) and right-side temporal (T4) electrodes (near to the location of the primary auditory cortex area), as they showed the strongest response to auditory stimuli compared to other electrodes.

After filtering the data, we computed the Hurst exponent and entropy of EEG signals based on Rescaled Range Analysis [24], and approximate entropy [25] techniques using our written MATLAB codes.

### Statistical analysis

Mean values for the dependent variable (the Hurst exponent and approximate entropy of EEG signals) were compared across no stimulus, and stimulation conditions with a one-way fixed effect ANOVA. Mauchly's test (α = 0.05) was conducted in order to test for sphericity. In fact, Mauchly's sphericity test is a statistical test used to validate a repeated measures analysis of variance (ANOVA). Trend analysis was performed across conditions when ordered according to the properties of the auditory stimuli. For a repeated measures design, we used Omega squared (ω^2^) as an unbiased measure of the effect size suitable for small samples. For pairwise comparisons effect size, *r*, was used. It is noteworthy in order to have robust results, all assumptions in case of each statistical test were fulfilled.
